# Evaluating Environmental Persistence and Disinfection of the Ebola Virus Makona Variant

**DOI:** 10.3390/v7041975

**Published:** 2015-04-14

**Authors:** Bradley W. M. Cook, Todd A. Cutts, Aidan M. Nikiforuk, Philip Guillaume Poliquin, Deborah A. Court, James E. Strong, Steven S. Theriault

**Affiliations:** 1 Applied Biosafety Research Program, Canadian Science Centre for Human and Animal Health and J. C. Wilt Infectious Diseases Research Centre, Public Health Agency of Canada, 1015 Arlington Street, Winnipeg, MB R3E 3P6, Canada and 745 Logan Street, Winnipeg, MB R3E 3L5, Canada; E-Mails: bradley.cook@phac-aspc.gc.ca (B.W.M.C.); todd.cutts@phac-aspc.gc.ca (T.A.C.); aidan.nikiforuk@phac-aspc.gc.ca (A.M.N.); 2 Department of Microbiology, The University of Manitoba, Winnipeg, MB R3T 2N2, Canada; E-Mail: dcourt@cc.umanitoba.ca; 3 Diagnostics Unit, Special Pathogens Program, Canadian Science Centre for Human and Animal Health, Public Health Agency of Canada, 1015 Arlington Street, Winnipeg, MB R3E 3P6, Canada; E-Mails: guillaume.poliquin@phac-aspc.gc.ca (P.G.P.); jim.strong@phac-aspc.gc.ca (J.E.S.); 4 Department of Pediatrics and Child Health, The University of Manitoba, Winnipeg, MB R3T 2N2, Canada

**Keywords:** filovirus, Ebola virus, ebolavirus, Makona, environment, disinfection, sodium hypochlorite, ethanol, outbreak management, biosafety

## Abstract

Background: The current disease outbreak caused by the Ebola virus Makona variant (EBOV/Mak) has led to unprecedented morbidity and lethality given its geographic reach and sustained transmission. Sodium hypochlorite and ethanol are well-accepted decontamination agents, however little published evidence supports the selection of appropriate concentrations and contact times. The present study addresses the environmental robustness of EBOV/Mak and evaluates the effectiveness of sodium hypochlorite and ethanol as disinfectants. Methods: EBOV/Mak was suspended in a simulated organic soil load and dried onto surfaces. Viability was measured at 1 hour, 24 hours, 72 hours, and 192 hours. For the evaluation of disinfectants, EBOV/Mak in a simulated organic soil was dried onto stainless steel carriers and disinfected with 0.01% (v/v), 0.1% (v/v), 0.5% (v/v) and 1% (v/v) sodium hypochlorite solutions or 67% (v/v) ethanol at contact times of 1, 5 or 10 minutes. Results: EBOV/Mak persisted longer on steel and plastic surfaces (192 hours) than cotton (<24 hours). Dilute sodium hypochlorite (0.01% and 0.1%) showed little antiviral action, whereas 0.5% and 1% sodium hypochlorite solutions demonstrated recoverable virus at one minute but sterilized surfaces in five minutes. Disinfection with 67% ethanol did not fully clear infectious virions from 3/9 carriers at 1 minute but sterilized all carriers at 5 and 10 minutes. Conclusions: Sodium hypochlorite and ethanol effectively decontaminate EBOV/Mak suspended in a simulated organic load; however, selection of concentration and contact time proves critical.

## 1. Introduction

Ebola virus (EBOV), species *Zaire ebolavirus* and family *Filoviridae*, has caused episodic disease outbreaks with high lethality in Africa since 1976. The current Ebola virus Makona variant (EBOV/Mak) epidemic originated from Guéckédou, Guinea in early December 2013 and extensive human-to-human transmission has caused the virus to spread across six African nations. Additionally, Ebola virus disease (EVD) patients have been medically evacuated to Europe and the United States, nosocomial transmission has occurred in both Spain and the United Sates [1]. The scale of the current epidemic has heightened the need for effective environmental decontamination procedures, as the geographic spread and caseload places a strain on previously successful infection control measures. The development and implementation of effective infection control protocols requires evidence-based knowledge about the transmission of EBOV/Mak, including the persistence of the virus on environmental surfaces and the effectiveness of common disinfectants against the virus.

The field of filovirus decontamination contains limited published literature. The role of gamma irradiation and ultraviolet exposure has been relatively well studied [2,3,4]. These methods are evidently impractical in clinical settings due to cost and difficulty of application. The ability of EBOV to persist in the environment is poorly defined. A number of physical, chemical and biological factors determine the persistence of a virus in the environment. EBOV should theoretically persist poorly in the environment as desiccation of viral lipid envelopes typically reduces infectivity [5]. However, a growing body of evidence challenges the assumption of EBOV’s fragile nature [6,7]. Two physical properties that are thought to contribute significantly to viral persistence are the nature of suspension sheltering a virus and the surface that suspension occupies [8]. It has been shown that surface type affects length of EBOV survival, though results have thus far been inconsistent [6,7]. Additionally, the material suspending the virus appears to be important, as EBOV suspended in serum persisted in the environment for up to 46 days—approximately 10 times longer than EBOV suspended in tissue culture media [7].

It is not yet known how porous surfaces impact EBOV persistence. The extended survival of EBOV suspended in serum suggests that enveloped viruses may persist better in organic solutions with high protein concentrations than protein deficient ones [6,7]. Patients suffering from advanced EVD commonly excrete vomitus and diarrhea, fluids with great protein complexity [9]. Therefore, we hypothesize that excreta of an EBOV-infected patient enables viral persistence in the environment and subsequently the potential for fomite transmission. To test this notion, EBOV/Mak virus was suspended in a surrogate complex organic soil load designed to mimic the protein secretions of a symptomatic EVD patient. The environmental persistence of EBOV/Mak in this organic soil load was then challenged on personal protective equipment (PPE) and stainless steel surfaces common to clinical settings.

The issue of EBOV disinfection also requires additional examination, given its importance to outbreak management and relationship to environmental persistence. If an organic soil load was demonstrated to favor the persistence of EBOV/Mak in the tested environments then it could also affect the virus’s susceptibility to chemical inactivation. Governing bodies recommend disinfection of EBOV-contaminated surfaces with a 0.5% sodium hypochlorite solution [10]; however no accessible experimental data supports this concentration selection or establishes a contact time. Furthermore, to our knowledge no formal studies have tested the efficacy of alcohol-based hand sanitizer (a popular hospital antiseptic), as a disinfectant against EBOV. We designed experiments to quantify the action of sodium hypochlorite (0.01%, 0.5%, 0.1% and 1% (v/v) solutions) and 67% (v/v) ethanol as disinfectants against EBOV/Mak over select contact times.

Overall, our study aims to elucidate the environmental persistence of EBOV/Mak in an organic soil load on clinically relevant surfaces and outline the effectiveness of sodium hypochlorite and ethanol at inactivating EBOV.

## 2. Materials and Methods

### 2.1. Propagation of EBOV/Mak Virus

Serum isolated from a sixteen-year-old female patient (C05) from Guéckédou, Guinea diagnosed with EVD caused by EBOV/Mak (Ebola virus/H.sapiens-tc/GIN/2014/Makona-C05, GenBank: KJ660348) was blind passaged in the Containment Level 4 (CL-4) laboratory (Canadian Science Centre for Human and Animal Health (CSCHAH), Winnipeg, MB, Canada). Vero E6 cells provided by the Centers for Disease Control and Prevention (Atlanta, GA, USA) were maintained in Dulbecco’s Modified Essential Medium (DMEM) (HyClone, Ottawa, Ontario, Canada) supplemented with Fetal Bovine serum (FBS) (Gibco, Burlington, Ontario, Canada) and 1% penicillin and streptomycin (Gibco). One day prior to infection, Vero E6 cells were sub-cultured to 80%–90% confluence in a T150 flask (Corning, Ottawa, Ontario, Canada), 24 hours prior to infection. The cells were infected with serum from patient C05 and incubated for 1 hour at 37 °C/5% CO_2_. Virus infection medium (2% FBS and 1% antibiotics in DMEM) was then added and the flasks were incubated. Once 70%–80% CPE developed at approximately 9 days, EBOV/Mak was harvested by centrifugation at 5000 × *g* for 10 minutes and aliquots were stored in liquid nitrogen as a passage one (P1) stock.

A P1 stock was removed from liquid nitrogen and used to infect, 9-T150 flasks (MOI = 0.05) of 80%–90% confluent Vero E6 cells. After absorption for 1 hour, virus infection medium was added and the flasks were re-incubated at 37 °C/5% CO_2_. When 70%–80% CPE developed, the flasks were frozen overnight at −70 °C. After thawing, cellular debris was removed by centrifugation at 5000 × *g* for 10 minutes followed by additional centrifugation (108,000 × *g*) on a 20% sucrose cushion for two hours. Concentrated virus was re-suspended in virus infection medium, stored and titrated. EBOV/Mak from two separate P2 stocks was used for these experiments (P2A and P2B), as indicated in the results section.

### 2.2. Determination of the Environmental Persistence of EBOV/Mak

Environmental Surfaces: lightly-scratched, stainless steel disks were prepared as 0.5 cm^2^ carriers (fabricated at CSCHAH). The carriers were sterilized by submersion in 70% ethanol and heat-treatment at 160 °C for 2 hours. Hospital-grade PPE, including: surgical mask (N95 Pleats Plus, AOSafety, Indianapolis, Indiana, USA), cotton gown (Medline, Oakville, Ontario, Canada) and waterproof plastic gown (FiveStar, Markham, Ontario, Canada) were prepared as 0.5 cm^2^ carriers and sterilized with 1 MRAD of gamma irradiation. EBOV/Mak was inoculated into a simulated organic soil load following the ASTM International (formerly American Society of Testing and Materials) Quantitative Carrier Testing 2 international standard [11,12]. Specifically, this consisted of: 106.25 µL of EBOV/Mak (P = 2 stock), 12.5 µL 5% BSA (Sigma, Oakville, Ontario, Canada, 17.5 µL 5% tryptone (Becton Dickinson, Mississauga, Ontario, Canada), and 50 µL 0.4% mucin (Sigma). The design of the organic soil load intentionally mimics residual bodily fluids serving as a general standard for testing [11]. Ten microliters of virus-soil load mixture was deposited onto each carrier with a positive displacement pipette (Eppendorf, Mississauga, Ontario, Canada) and allowed to dry in a biological safety cabinet for 1 hour at 21.5 °C and 30% relative humidity. Samples were recovered from the carriers at each time point (0, 1, 24, 72, and 192 hours) by pipetting with 1 mL of virus infection medium over the surface of the carrier. This wash was then stored at −70 °C until quantification by TCID_50_ assay. Three technical repetitions were performed for each surface at every time point (n = 3).

### 2.3. Fifty-Percent Tissue Culture Infectious Dose (TCID_50_/mL) Assay

Vero E6 cells were grown overnight to 80%–90% confluence in 96-well plates. Samples were thawed and 10-fold serially diluted, each dilution was added in replicates of 5 to cell monolayers for 1 hour at 37 °C/5% CO_2_. Following absorption, 150 µL of viral infection medium was added to each well for a final volume of 200 µL. The plates were incubated for 14 days post-infection, scored for CPE and TCID_50_/mL was calculated by the Reed and Muench method [13].

### 2.4. Evaluating the Activity of Four Sodium Hypochlorite Concentrations and 67% Ethanol against EBOV/Mak

Sodium hypochlorite: On the day of each assay, fresh bleach (Imperial sanitizer IMP750-1) containing 10.8% sodium hypochlorite and 10.3% available chlorine was diluted to final concentrations of 1%, 0.5%, 0.1%, and 0.01% v/v sodium hypochlorite in hard water (containing 0.04% w/v calcium carbonate). Concurrently, a 1% v/v solution of sodium thiosulfate in DMEM was made to neutralize the activity of sodium hypochlorite at an established contact time. EBOV/Mak-soil load mixtures were prepared and deposited on steel carriers, as described above. Fifty µL of each sodium hypochlorite concentration was added to the carriers and neutralized with 950 µL of 1% sodium thiosulfate at (1, 5, and 10 minutes). Time point 0 served as a positive control where 1000 µL of 1% sodium thiosulfate was added to the dry carriers. Five hundred microliters of each sample was removed for titration by TCID_50_.

Previous use of 1% sodium hypochlorite and 1% sodium thiosulfate demonstrated cytotoxicity to Vero E6 cells when undiluted (10^0^). This made the 10^−1^ dilution the limit of virus detection by TCID_50_ assay, as we could not determine whether the observed CPE at the 10^0^ was due to chemical damage or viral infection. To confirm an absolute kill at the 5 and 10-minute contact times for the 0.5% and 1% sodium hypochlorite samples the remaining 500 µL of each 10^0^ dilution was pooled in replicates of three. These three 1.5 mL solutions were used to infect T150 flasks of Vero E6 cells, as the extensive dilution overcomes chemical toxicity. These flasks were incubated for 14 days and observed for CPE. No viral infection in these flasks would confirm absolute kill of EBOV/Mak.

Ethanol: Fresh solutions of 67% ethanol were prepared in hard water (containing 0.04% w/v calcium carbonate). EBOV/Mak-soil load mixtures were deposited on steel carriers and dried, as described earlier. To evaluate disinfection, 50 µL of 67% ethanol was added to the carriers and neutralized with 950 µL of virus infection medium. As a control, no ethanol was added to time point 0; instead the carriers were washed with 1000 µL of virus infection medium. After gentle pipetting, 500 µL was removed for titrations. As neutralized ethanol caused no cytotoxicity the TCID_50_ assay possessed a full range of detection (10^0^–10^8^). For the tested disinfectants three biological replicates of 3 technical repetitions were performed at every time point (n = 9).

### 2.5. Statistical Analysis

All TCID_50_ calculations, averaging and determination of standard deviations were performed in Microsoft Excel 2011. GraphPad (Prism 5) software was used to generate figures and calculate the standard error of the mean, which was used to make positive and negative error bars. Statistical analysis by the students T-test and analysis of variance (ANOVA) were performed using the SPSS statistical software suite (IBM, New York, NY, USA).

Non-linear regression analysis of EBOV/Mak persistence data was performed using the one phase decay equation of GraphPad (Prism 5). The accuracy of the models’ was determined by the R2 value and replicates test. The one phase decay equation ($y=\left(y_{0}-p\right)\times e^{\left(-\kappa \times t\right)}+p$) was solved for time (t) when the model projected a one- (90%) and four- (99.99%) log_10_ reduction in viral titer.

## 3. Results

### 3.1. Environmental Stability of EBOV/Mak on Surfaces Common to Clinical Settings

**Figure 1:**
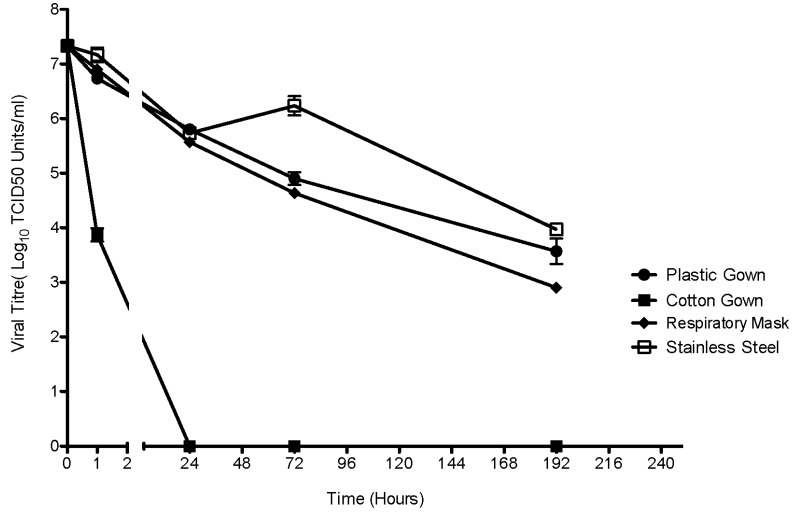
Environmental persistence of EBOV Makona (EBOV/Mak) suspended in an organic soil load. The robustness of high titre EBOV/Mak inoculums in an organic soil load was tested on 4 surfaces commonly used in health care environments at 21.5 °C and 30% relative humidity. Viral titres were measured per environmental carrier by the TCID_50_ assay. The error bars represent the standard error of the mean. Three technical repetitions were performed for each surface at every time point (n = 3).

EBOV/Mak suspended in an organic soil load showed environmental persistence on common hospital surfaces. Figure 1 illustrates the reduction of recoverable viral titer over time of environmental exposure. There was no significant difference in the initial viral inoculum of 7.3-log10 TCID_50_ units/mL (SD ± 0.34). The solid surfaces of the surgical mask, plastic gown and steel carriers allowed for prolonged viral survival. There was no significant difference (*p* = 0.058) in the viral titer at 192 hours between the surgical mask (2.9 log_10_ TCID_50_/mL) and plastic gown (3.6 log_10_ TCID_50_/mL). The virus persisted better on stainless steel carriers (4.0 log_10_ TCID_50_/mL) than on both plastic surfaces (3.2 log_10_ TCID_50_ units/mL, *p* = 0.033) over the same time period. On the rough textile surface of a cotton gown a 47% reduction in viral titer occurred after one hour of exposure, followed by complete inactivation at 24 hours. Non-permeable surfaces enabled substantially longer EBOV/Mak persistence than a permeable surface such as cotton. While there was relatively rapid inactivation of the virus on cotton, it is important to note that sufficient virus to cause infection was recoverable at the one-hour mark, the estimated infectious dose ranges between 1 and 10 virions [13]. Since the steel carriers allowed for the greatest amount of recoverable EBOV/Mak over time, this surface was selected for testing the efficacy of disinfectants. A non-linear regression model calculated the exponential rate of decay for EBOV/Mak virus on the tested surfaces (Table 1). The models show that the virus theoretically persists longest on stainless steel surfaces, a four-log reduction (99.99%) occurs after 365 hours of exposure. All of the models possess a high R^2^ value indicating closeness of fit. To additionally, examine the accuracy of the models a replicates test was performed, all of the models passed this test with the exception of stainless steel.

**Table 1:** Results of non-linear regression analysis for modeling the exponential decay of EBOV/Mak virus 7.3 TCID_50_/mL (t = 0) on the tested surfaces. The fitted models were utilized to calculate the time until one and four-log reductions in viral titer occur from the initial viral input.

Tested Surface	Decay Constant (ĸ)/1 Hour	Decay Constant (ĸ)/24 Hours	R^2^	One-Log Reduction (TCID_50_/mL)	Time (Hours)	Four-Log Reduction (TCID_50_/mL)	Time (Hours)
Plastic Gown	0.0117	0.28	0.96	6.3	24	3.3	285
Cotton Gown	0.6395	15.35	0.99	6.3	0.24	3.3	1.24
Stainless Steel	0.0091	0.22	0.90	6.3	30	3.3	365
Respiratory Mask	0.0113	0.27	0.97	6.3	20	3.3	147

### 3.2. Disinfection of EBOV/Mak with Sodium Hypochlorite

**Figure 2:**
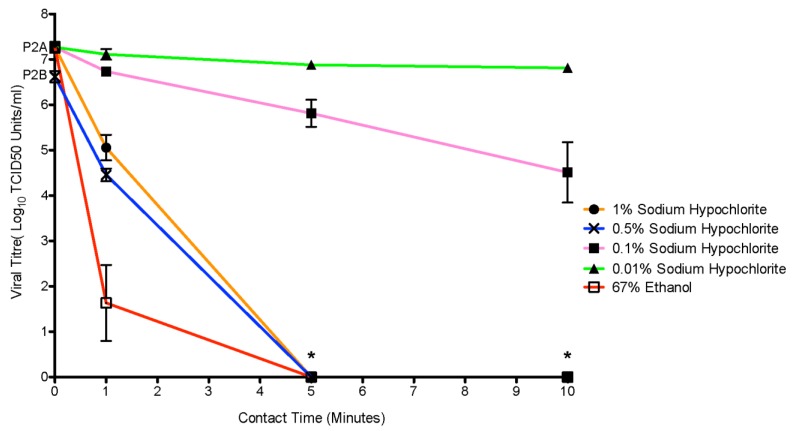
Efficacy of the standard disinfectants sodium hypochlorite and ethanol against EBOV Makona (EBOV/Mak) in an organic soil load**.** Various concentrations of v/v sodium hypochlorite (0.01%, 0.5%, 0.1%, 1%) and 67% ethanol were tested over contact times of 1 minute, 5 minutes and 10 minutes. Recovered viral titres were measured per/mL by the TCID_50_ assay. The asterisks (*) denote where a limit of detection for the TCID_50_ assay was overcome for 0.5% and 1% sodium hypochlorite samples. In order to confirm complete kill at the 10^0^ dilutions the samples were pooled in three groups of three (n = 9) and used to infect T150 flasks. Complete kill of EBOV/Mak with 0.5% and 1% sodium hypochlorite treatment was confirmed by this method for the 5 and 10 minute contact times. The error bars represent the standard error of the mean. For the tested disinfectants three biological replicates of 3 technical repetitions were performed at every time point (n = 9).

The ability of (0.01%, 0.1%, 0.5% and 1%) concentrations of sodium hypochlorite to inactivate suspended EBOV/Mak on steel carriers was tested at three contact times (Figure 2). Initial virus inoculum, was an average of 7.3 log_10_ (SD +/− 0.34) across experimental replicates using the P2A stock; namely the 0.01%, 0.1% and 1% experiments. The 0.5% experiment was conducted with the P2B virus stock, with a lower input load (6.6 SD +/− 0.3 TCID_50_ units/mL). At the lowest tested sodium hypochlorite concentration (0.01%) no significant reduction in viral titer was observed (*p* = 0.079). A 0.1% sodium hypochlorite solution proved to be partially effective against the virus at a 10-minute contact time but 3.7 log_10_ (SD +/− 2.0) viable virus particles remained. The 0.5% and 1% sodium hypochlorite solutions performed similarly. At both concentrations, viable virus was recoverable at one minute (4.4 log_10_ SD +/− 0.4, 5.1 log_10_ SD +/− 0.85, respectively), while all stainless steel carriers were sterile following a five-minute contact time. The higher viral load observed at the 1 minute mark with the 1% concentration of sodium hypochlorite compared to the 0.5% solution is likely a reflection of lower input virus on the steel carriers used for the 0.5% experiments, as the average reduction (2.2 log_10_ TCID_50_ units/mL) was the same for both concentrations. Infection of T150 flasks of Vero E6 with pooled 10^0^ dilutions (n = 9) of the 0.5% and 1% sodium hypochlorite samples confirmed absolute kill. The efficiency of EBOV/Mak disinfection positively correlated to the concentration of sodium hypochlorite and length of contact times. Within the test conditions, higher concentrations of sodium hypochlorite at longer contact times achieved greater disinfection.

### 3.3. Disinfection of EBOV/Mak with 67% Ethanol

We tested the effectiveness of 67% ethanol, a common ingredient of gel-based hand sanitizers and a clinical disinfectant, at inactivating EBOV/Mak in a simulated organic soil load on steel carriers. Figure 2 demonstrates that 67% ethanol was able to inactivate the virus on all but 3/9 (33%) of carriers after one minute. No recoverable virus was found after 5 or 10 minutes of contact time.

## 4. Discussion

The unprecedented scale of the 2014 EVD outbreak has global implications with the international movement of patients and the return of infected health care workers to their home nations from Western Africa [1]. As health care organizations around the globe prepare their personnel for a domestic or international EBOV response, they require current high quality evidence to devise environmental decontamination and outbreak management protocols. This study provides previously undocumented information on the environmental persistence and disinfection of EBOV in controlled hospital settings.

EBOV/Mak in an organic soil load survived for lengthy periods of time on the examined surfaces. This finding agrees with previously reported EBOV environmental persistence data [6,7] suggesting that EBOV can tolerate long-term environmental exposure dependent on the conditions. The prolonged persistence of EBOV/Mak was supported by the reliable recovery of infectious virus from stainless steel and plastic-covered surfaces at 192 hours. The study did not extend beyond eight days to focus on the need to cleanse PPE after contamination especially before re-use. The persistence of EBOV/Mak on a textile cotton surface differed greatly from the solid surfaces as the virus underwent rapid inactivation with a steep kill curve. A similar scenario was observed for other enveloped viruses indicating that porous surfaces negatively affect their viability [14]. However, since virus was recovered near one hour post-inoculation, this indicates that recently exposed, linen and steel surfaces constitute an important nosocomial risk. Overall, EBOV/Mak persisted on the surface of all the tested materials including common elements of PPE for over an hour. Therefore, materials suspected of contamination should be disinfected immediately, with appropriate attention given to the applied concentration of disinfectant and contact time.

During previous EVD outbreaks, health care professionals applied sodium hypochlorite to successfully decontaminate clinical instruments, laboratory materials and personal protective equipment [15]. The World Health Organization (WHO) currently recommends the use of a 0.5% available chlorine solution to disinfect surfaces contaminated with EBOV [10]. This represents a change from previous guidelines that recommended a 0.5% solution for decontamination of bodies and excreta and a 0.05% solution for contaminated surfaces and equipment [16]. Our results (1%, 0.5%, 0.1% and 0.01%) suggest that the current WHO recommendation of approximately 0.5% sodium hypochlorite does have effective antiviral properties when applied to a simulated organic load, with the caveat that sufficient contact time must be provided. Even the highest tested concentration of sodium hypochlorite (1%) did not sterilize EBOV/Mak infected surfaces at one minute of contact time. Therefore, successful disinfection with sodium hypochlorite dually depends on concentration selection and contact time.

In some clinical situations ethanol diluted in water or as an ingredient of hand sanitizer is preferred to sodium hypochlorite as a disinfectant, as it does not cause corrosive damage to sensitive equipment and skin [17]. There is no clear recommendation regarding the most effective concentration of ethanol to be used in such products. The Food and Drug Administration suggests a 60% to 95% range as appropriate but do not establish a formal guideline [18,19]. A previous study focused on surrogate virus testing found similar virucidal activity across a range of ethanol concentrations (58%–75%) [20]. We selected 67% ethanol as a mid-range concentration hoping that it reflects a wide range of commercial products. The sterilization of 6/9 EBOV-Mak contaminated steel carriers with 67% ethanol at 1 minute advocates that ethanol outperforms sodium hypochlorite at inactivating EBOV/Mak. However, the substantial action of ethanol should not deter the use of proper PPE or sodium hypochlorite in managing outbreaks of EBOV. It does support that universal precautions such as hand washing with alcohol-based sanitizers provide some protection, even from high profile filoviruses. This information can help reduce stress to frontline health care workers who may, inadvertently, become exposed to an EBOV infected patient prior to diagnosis.

Limitations of the present study include a fairly wide choice of contact times (1, 5, and 10 minutes). We conducted the experiments under the chosen conditions in order to generate a range of data and are currently working to identify ideal contact times for disinfection of several EBOV variants with a variety of chemical disinfectants (*i.e.*, Micro-Chem Plus). The Makona variant shows 97% nucleotide similarity to other EBOV variants, including Kikwit and Yambuku [21]. Thus, our future planned experiments will determine if there are any functional differences associated with environmental persistence or disinfection characteristics. Furthermore, testing system does not account for the full range of conditions that affect persistence, such as changes in humidity, temperature or exposure to UV light. In regards to rate of decay calculations, all of the models passed with a single exception. The stainless steel model failed because the distance from the line of exponential decay and the data points exceeded the variation between data points. Two factors likely contribute to this discrepancy: little variation within replicates of the experimental data and the recovery of more viral particles at 72 hours then at 24 hours. Completion of further experimental replicates would ensure greater accuracy of the model. Despite these limitations, the present testing conditions (*i.e.*, high viral titer and an organic soil load) are stringent but clinically appropriate, thus providing confidence in the applicability and safety of conclusions.

Suspension of high titer EBOV/Mak virus in a medium simulating the secretions of an infected patient supported persistence of the virus for at least 192 hours (eight days) on the surface of common healthcare equipment. This makes rigorous decontamination procedures vital to outbreak management. The use of sodium hypochlorite as a primary disinfectant appears generally effective; however, the concentration of sodium hypochlorite and contact time heavily influence antiviral action. A similar trend was observed in the challenge of EBOV/Mak with 67% ethanol as viral titer decreased over the duration of contact time. Health care workers should take precaution to not leave EBOV/Mak contaminated surfaces unmanaged and when faced with decontaminating PPE or other equipment should consider an appropriate selection of disinfectant as well as contact time. This study of EBOV/Mak suspended in an organic soil load concludes that a 0.5% or 1% solution of sodium hypochlorite and 67% ethanol completely sterilized EBOV/Mak contaminated stainless steel surfaces following five minutes of contact. Overall, these findings suggest that the environmental persistence of infectious EBOV/Mak on clinical surfaces is a substantial risk, even after several days, and that choice of disinfectant concentration and contact time are important factors in remediating contaminated areas.
